# Antibacterial potential of streptomycete strains from Antarctic soils

**DOI:** 10.1080/13102818.2014.947066

**Published:** 2014-10-16

**Authors:** Marta Encheva-Malinova, Mariya Stoyanova, Hristina Avramova, Yanitsa Pavlova, Blagovesta Gocheva, Iskra Ivanova, Penka Moncheva

**Affiliations:** ^a^ Faculty of Biology, Sofia University ‘St. Kliment Ohridski’, Sofia, Bulgaria; ^b^ Institute of Soil Science, Agrotechnologies and Plant Protection ‘Nikola Poushkarov’, Sofia, Bulgaria

**Keywords:** antibacterial activity, Antarctic, PCR-amplification, phytopathogenic bacteria, streptomycetes

## Abstract

The exploration of habitats with unusual environment and poorly explored areas such as Antarctica is one of the strategies for discovery of new biologically active substances and/or new producers. The aim of this study was to identify the actinomycetes isolated from the soils of the island Livingston – Antarctica and to investigate their potential to synthesize antibacterial agents against phytopathogens. Twenty-three actinomycete strains were the object of this study. Using PCR (polymerase chain reaction) amplification all strains were affiliated to genus *Streptomyces*.

The sequencing of the 16S rRNA for three of the strains showed greatest similarity to *Streptomyces tendae* for one of them, and revealed that the other strains had closest relations to streptomycetes isolated from anthropogenically unaltered regions including Antarctica.

The isolates were studied for production of antibacterial substances both by molecular and culture methods. PCR targeting specific biosynthetic genes involved in the production of some groups of antibiotics was performed. The screening showed that all strains possessed the gene for Type-II polyketide synthase, 11 strains – for non-ribosomal peptide synthetase; 6 strains – for polyene antibiotics; and 4 strains – for glycopeptide antibiotics. The production of antibacterial substances by the strains was tested in vitro against phytopathogenic bacteria. The strains differed in the number of inhibited test – bacteria and in their spectrum of action. Four strains showed a wide range of activity against Gram-positive and Gram-negative phytopathogens.

The results obtained revealed that the Antarctic soils are potential source for isolation of streptomycetes producing antibiotics from different groups.

## Introduction

Antarctica is the southernmost continent and is surrounded by the southern waters of the World Ocean. It is the coldest, driest and windiest continent; approximately 98% of it is covered by ice. Despite the harsh and unique environmental conditions, a greater than expected organism diversity was found – including a number of endemic and yet unknown species.

By molecular methods it was demonstrated that most of the isolated Antarctic prokaryotes are new species. Streptomycetes are Gram-positive, filamentous typical soil bacteria with high content of G + C and belong to the group of the actinomycetes which are relatively less investigated as a component of microbial communities in Antarctica, but there are studies that demonstrate their ability to synthesize biologically active substances of different nature.[1–8] The genus *Streptomyces* is composed of a large number of species many of which synthesize a broad spectrum of secondary metabolites. Several studies have been focused on identifying biocontrol agents which could be used as an alternative to agrochemicals in plant protection.[9]

The detection of gene sequences involved in the synthesis of secondary metabolites by different microorganisms, including actinomycetes, made possible the development of rapid screening techniques, which are easy to implement in the study of the biosynthetic potential of the strains from different habitats.[4,10–13]

Antarctic soils are less-investigated ecological systems and are a potential source for discovery of actinomycetes with previously unknown metabolic capacity with respect to the synthesis of biologically active substances including antibiotics. This study aims to identify actinomycete strains isolated from soils of Livingston Island, Antarctica, and to investigate their potential to synthesize antibacterial agents against phytopathogenic bacteria.

## Materials and methods

### Strains

Twenty-three actinomycete strains originating from soils of Livingston Island, Antarctica were the object of this study. Seventeen of the strains were isolated in two different years from five Antarctic soils and six were from the Antarctic collection of the Department of General and Industrial Microbiology of Sofia University ‘St. Kliment Ohridski’, Bulgaria.

### DNA extraction

DNA was extracted from 48 h cultures grown in YEME (yeast extract-malt extract) medium on a rotary shaker at 220 rpm, 28 °C using NucleoSpin® Tissue (MACHEREY-NAGEL) kit according to the manufacturer's instructions. DNA from *Streptomyces hygroscopicus* 155 was used as control.[14]

### PCR-amplifications of 16S rDNA

The amplification of 16S rDNA was performed by two pairs of *Streptomyces* genus-specific primers: StrepB/StrepE and StrepB/StrepF in conditions described by the authors.[15] The amplification was done in a total volume of 25 μL containing (final concentration): 0.5x *Taq* Master Mix RED (VWR), 4 pmol of each primer (STS Ltd.) and 100 ng DNA.

The PCR products were analysed on a 1% TBE (Tris-borate-EDTA) agarose gel, stained with ethidium bromide at 100 V for 30 min, visualized under ultraviolet light transillumination and photographed with Gel Documentation and Analysis System GenoMini (VWR).

For the sequence analysis amplification of 16S rDNA was carried out by the universal primers 9f (5′-GAGTTTGATCCTGGCTCAG-3′) and 1542r (5′-AGAAAGGAGGTGATCCAGCC-3′).[16] The amplification was performed in a total volume of 50 μL, containing (as final concentration) 38 μL sterile water; 5 μL 10х PCR buffer; 1.8 μL MgCl_2_; 0.75 μL dNTP; 0.16 μL *Taq* DNA polymerase; 1 μL from each of the primers (9f and 1542r); 1 μL of the DNA samples at the conditions according to Stackebrandt et al.[16]

### Sequencing of the PCR products of 16S rDNA

The PCR products obtained with the primer pair 9f/1542r were sequenced for 3 of the strains by the company Macrogen (the Netherlands). The results were compared with the database on NCBI, analysed with the program BLAST 2 and visualized with the program MEGA 5.

### Screening for antibiotic biosynthetic genes

The PCR screening for the biosynthetic genes involved in the production of antibiotics from different groups was based on the amplification with specific primers. All amplifications were carried in a total volume of 25 μL, containing (final concentration): 0.5x Taq Master Mix RED (VWR), 4 pmol of each primer (STS Ltd.) and 100 ng DNA.

PCR screening for the non-ribosomal peptide synthetase (NRPS) genes involved in the biosynthesis of biologically active peptide compounds was performed by amplification with primers A3 and A7R under the conditions described by the authors.[11]

PCR screening for the biosynthetic genes for Type-II polyketides (PKS-II) and glycopeptide antibiotics was performed using the primer pairs ARO-PKS-F/ARO-PKS-R and Foxy/Roxy, respectively,[12] and the reactions were performed according to Mavengere.[4]

PCR screening for the gene coding a polyene-specific enzyme cytochrome P450 hydroxylase (CYP) was carried out by a pair of degenerative primers I and II according to the author.[13]

### In vitro screening of antibacterial activity

The antibacterial activity of the streptomycete strains was assayed by the diffusion agar plug method measuring the diameter of the inhibition zones. The agar plugs were prepared by streptomycetes cultures grown on ISP Medium 4. Potato dextrose agar (Oxoid) for phytopathogenic bacteria and Antibiotic medium 2 (Difco) for *Bacillus subtilis* and *Escherichia coli* were previously inoculated with 1.5.10^7^ CFU/ml (0.5 McFarland standards) of 24 h test-bacterial suspensions. The seeded plates with the agar streptomycete plugs were cultivated at 28 °C for 48 h. The used test bacteria were shown in Table 1. All experiments were performed in triplicate.

**Table 1.** Antibacterial activity of Antarctic streptomycetes.

	Antibacterial activity (inhibition zone, mm)													
	Streptomycete strains													
Test-bacteria	2M	5M	6M	7M	9M	10M	13M	39	2	4	6	8	10–4	11
*Bacillus subtilis* ATCC^1^ 6633	27	–	18	33	–	–	40	10	24	34	29	30	37	10
*Clavibacter michiganensis* NBIMCC^2^ 2425	38	13	26	36	9	10	43	–	–	–	27	25	23	13
*Burkholderia cepacia* 23 (onion isolate)^3^	–	–	–	–	–	–	–	–	–	–	–	–	–	–
*Burkholderia gladioli* 31(onion isolate)^3^	–	–	–	–	–	–	–	–	–	–	–	–	–	–
*Burkholderia pyrrocinia* 27 (onion isolate)^3^	–	–	–	–	–	–	–	–	–	–	–	–	–	–
*Erwinia amylowora* NBIMCC 8488 (pear isolate)	–	–	–	–	–	–	–	–	–	–	–	–	–	–
*Escherichia coli* NBIMCC 3397	–	–	–	–	–	–	–	–	–	–	–	–	–	–
*Xanthomonas euvesicatoria* 11t (tomato isolate)^3^	21	–	15	25	–	–	32	–	–	–	–	11	–	–
*Xanthomonas euvesicatoria* 52b (pepper isolate)^3^	17	–	–	18	–	–	40	–	–	–	11	12	–	–
*Xanthomonas gardneri* NBIMCC 8730	13	–	15	25	–	–	20	–	–	–	–	–	–	–
*Xanthomonas perforans* NBIMCC 8729	17	–	18	–	–	–	30	–	–	–	–	–	–	–
*Xanthomonas vesicatoria* 21t (tomato isolate)^3^	–	–	15	15	–	–	20	–	–	–	–	–	–	–

Note:^ 1^ATCC – American Type Culture Collection; ^2^NBIMCC – National Bank for Industrial Microorganisms and Cell Cultures (Bulgaria); ^3^The strains were kindly provided by prof. N. Bogatzevska (Institute of Soil Science, Agrotechnologies and Plant Protection ‘N. Poushkarov’, Sofia, Bulgaria).

## Results and discussion

**Figure 1.**
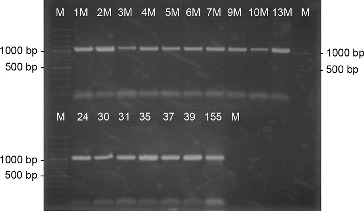
PCR-amplification of actinomycetes DNA with the genus-specific primer pair StrepB/StrepE. M- DNA Ladder (Fermentas); 155 – *S. hygroscopicus* 155; the number of each lane corresponds to the numbers of the strains.

**Figure 2.**
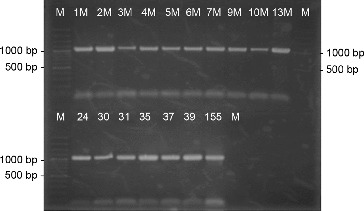
PCR-amplification of actinomycetes DNA with the genus-specific primer pair StrepB/StrepF. M- DNA Ladder (Fermentas); 155 - *S. hygroscopicus* 155; the number of each lane corresponds to the numbers of the strains.

On the basis of the morphological characteristics such as the well-developed aerial mycelium, the spore-bearing hyphae, and the spore surface, as well as the relatively easy isolation and maintenance, we assumed that the 23 actinomycete strains were members of the genus *Streptomyces*. Genus identification of all 23 strains was confirmed by PCR-amplification using two pairs of genus-specific primers. The amplified products by pair primers StrepB/ StrepE and StrepB/StrepF were about 520 and 1070 bp, respectively, which are specific for genus *Streptomyces* (Figures 1 and 2).[15,17] For part of the strains these results had already been obtained.[18]

Our results confirmed the wide distribution of the genus *Streptomyces* in Antarctic soils established by other authors.[2,4,19,20]

**Figure 3.**
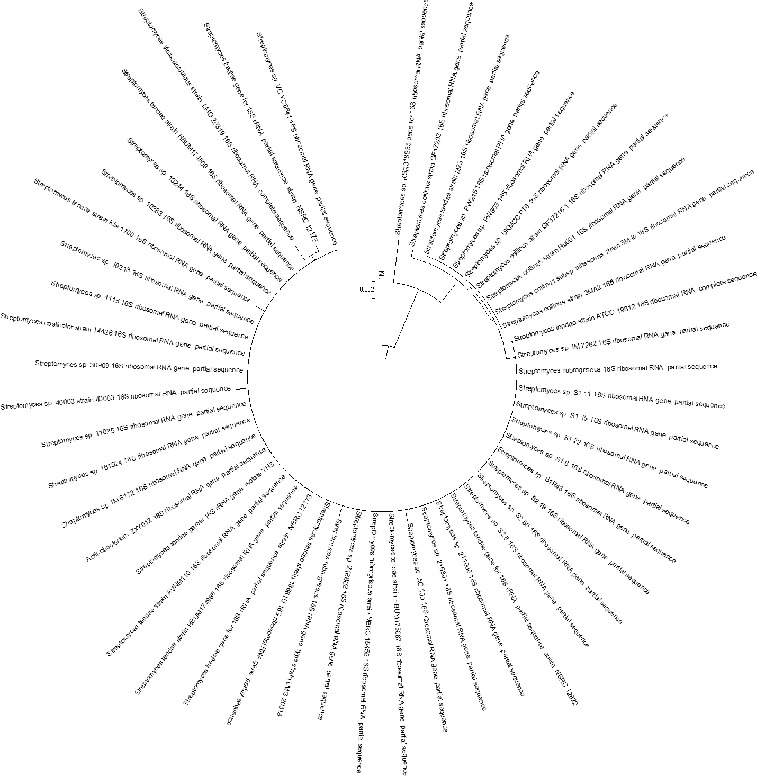
Phylogenetic tree showing the relation of strain 1M to other *Streptomyces* species based on the comparative analysis of the sequence of their 16S rDNA gene.

For three of the strains (1M, 2M and 7M), an attempt for species identification was made by amplification of their 16S rRNA gene with universal primers, followed by sequencing. The results of the sequence analysis are compared to the database on National Center for Biotechnology Information (NCBI) with the program BLAST. Strain 1M showed the closest relation to *S. tendae* (Figure 3). The comparative analysis of the sequences revealed closest relations of our strains to other *Streptomyces* strains isolated from anthropogenically unaltered regions including Antarctica.

**Figure 4.**
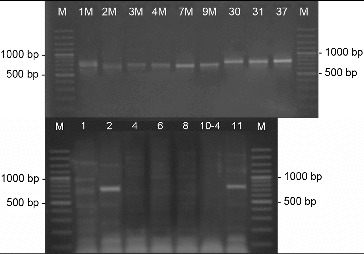
PCR-amplification of streptomycetes DNA for the gene involved in the synthesis of NRPS. M- DNA Ladder (Fermentas); the number of each lane corresponds to the numbers of the strains.

PCR screening for the biosynthetic genes involved in the production of various antibiotics showed that 11 of the analysed 23 strains possessed the gene for NRPS, forming a product with the expected length of 700–800 bp (Figure 4).[11]

The NRPS is a biosynthetic system involved in the synthesis of many important biologically active peptides produced by a range of microorganisms including the actinomycetes used in medicine, agriculture and biochemical studies. Using primers A3 and A7R, Ayuso-Sacido and Genilloud tested a large collection of 210 reference strains from all major families and genera of actinomycetes for NRPS.[11] The results showed that the target sequences were detected in most of the tested *Streptomyces* and *Nocardiaceae* strains (97% and 91%, respectively), but their occurrence varied very much in the other taxa. In our study it was determined that 56% of the tested Antarctic *Streptomyces* strains possessed the NRPS sequences.

**Figure 5.**
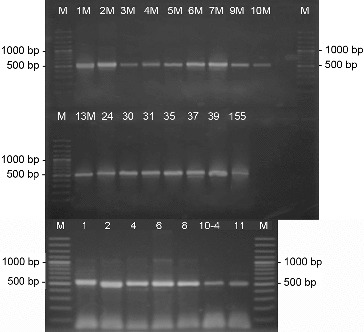
PCR-amplification of streptomycetes DNA for the genes involved in the synthesis of PKS-II. M- DNA Ladder (Fermentas); 155 - *S. hygroscopicus* 155; the number of each lane corresponds to the numbers of the strains.

The screening for PKS-II genes showed that all of the 23 analysed strains gave a positive result forming a product of about 500 bp (Figure 5). Type-II PKS are a family of multi-enzyme systems that catalyse the biosynthesis of therapeutically important aromatic polyketides, many of which produced by streptomycetes.[21] In a study of Wood et al.,[12] it was shown that the primer pair ARO-PKS-F/ARO-PKS-R for PKS-II genes, also used in our work, amplified a fragment of 492–630 bp depending on the bacterium tested. It should be noted however that the primers used may also amplify a KSα–KSβ fragment from genes involved in spore-pigment synthesis as it was determined for *S. avermitilis* ATCC 31267^T^, and [12] therefore our result had to be confirmed by other methods.

**Figure 6.**
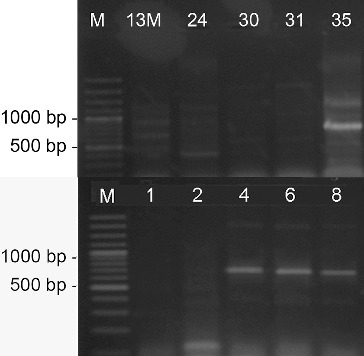
PCR-amplification of streptomycetes DNA for glycopeptide antibiotic genes. M- DNA Ladder (Fermentas); the number of each lane corresponds to the numbers of the strains.

The PCR screening for glycopeptides showed that four of the analysed strains showed a detectable amplification product with a length of 696 bp which is specific for the used primer pair FOXY/ROXY (Figure 6).[12] Glycopeptide antibiotics are a specific class of agents that inhibit cell wall synthesis in susceptible organisms and are synthesized by several actinomycete genera including *Streptomyces*. Analysing streptomycetes from Antarctica Mavengere did not detect the genes for glycopeptides.[4] This could mean that in this habitat a few streptomycete species synthesize glycopeptides or that representatives of other actinomycete genera are responsible for this.

**Figure 7.**
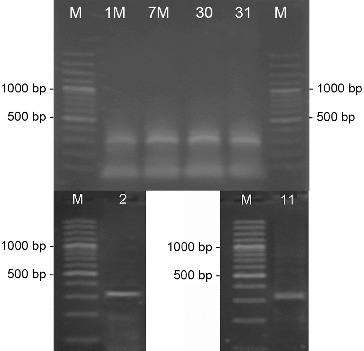
PCR-amplification of streptomycetes DNA for the gene involved in the synthesis of polyene antibiotics. M- DNA Ladder (Fermentas); the number of each lane corresponds to the numbers of the strains.

Of all streptomycete strains screened by PCR-amplification with degenerative primers, I and II, for six isolated products of about 350 bp were amplified, which correspond to the gene encoding polyene-specific enzyme CYP (cytochrome P450 hydroxylase) (Figure 7).

Polyene antibiotics are a class of antimicrobial compounds with fungicidal activity which are usually produced by actinomycetes of genus *Streptomyces*. The prevalence of fungal infections in recent decades, the low efficiency of the treatment of certain fungal infections, makes the search for new and more effective antifungal agents necessary. In the literature there is no information available on the potential of Antarctic streptomycetes to produce polyene antibiotics. Our study provides useful information for future research on this issue.

Based on the PCR screening performed, the strains can be separated into four groups according to their genetic potential to synthesize various bioactive substances. The widest biosynthetic potential had six of the strains possessing the genes for PKS-II, NRPS and CYP; five strains had the genes for PKS-II and NRPS; four strains possessed the genes involved in the synthesis of glycopeptide antibiotics and PKS-II; and eight strains seemed to possess only the genes for PKS-II. Most common among the studied Antarctic streptomycetes are the genes for PKS-II.

The streptomycete strains were screened for antibacterial activity against several Gram-positive and Gram-negative bacteria, most of which were phytopathogens (Table 1).

Eight of the strains were active against the test-bacteria under the particular conditions. None of the isolates showed activity against the phytopathogens *Burkholderia cepacia*, *B. gladioli*, *B. pyrrocinia*, *Erwinia amylowora* and against *E. coli*. The strains possessed different spectrum of action. Four of the strains (2М, 6М, 7М and 13М) demonstrated a broad spectrum of antibacterial activity and affected both Gram-positive and Gram-negative bacteria. Strain 13M showed the highest activity and inhibited the largest number of test-bacteria (Table 1).

The comparison of the data from the PCR screening for the biosynthetic genes involved in the production of antibiotics to the data from the *in vitro* screening for antibacterial activity showed that not all strains synthesized antimicrobial substances in *in vitro* conditions even if they had genes for them, particularly 1M, 3M, 4M and 35. It is interesting that strains 3M, 4M, and 35 belong to the groups encoding two types of bioactive substances (NRPS/PKS-II and glycopeptides/PKS-II) and strain 1M has the broadest genetic potential for antibiotic synthesis (PKS-II/NRPS/polyenes). The reasons for this discrepancy might be different – inhibition of the expression of these genes, unfavourable for antibiotic synthesis cultivation conditions (composition of culture medium, cultivation temperature, etc.), and were not a subject of the present work. At the same time strain 13M which had the broadest spectrum of antimicrobial activity *in vitro* and strain 6M also with good activity possessed only the genes for PKS-II.

Since the strains with greatest antimicrobial activity possess either PKS-II/NRPS/polyene, PKS-II/NRPS or only PKS-II genes, no clear statement of the nature of the particular substances responsible for this activity can be made. This study presents an initial screening for the biocontrol potential of the Antarctic streptomycetes and further research is needed to clarify the specific molecules involved in this process.

## Conclusions

Streptomycetes are an abundant group of soil microorganisms in Antarctica which was proved by cultural and molecular methods. All of our bioactive strains were proved to belong to the genus *Streptomyces*. The Antarctic streptomycetes show a good potential to produce different biologically active substances active against phytopathogenic bacteria.
